# Caregiver-perceived racial discrimination is associated with diverse mental health outcomes in Aboriginal and Torres Strait Islander children aged 7–12 years

**DOI:** 10.1186/s12939-019-1045-8

**Published:** 2019-09-06

**Authors:** Leah Cave, Matthew N. Cooper, Stephen R. Zubrick, Carrington C. J. Shepherd

**Affiliations:** 1 Telethon Kids Institute, The University of Western Australia, PO Box 855, West Perth, Western Australia 6872 Australia; 2 School of Population and Global Health, The University of Western Australia, 35 Stirling Highway, Perth, Western Australia 6009 Australia; 3 Centre for Child Health Research, The University of Western Australia, 35 Stirling Highway, Perth, Western Australia 6009 Australia; 4 Ngangk Yira Research Centre for Aboriginal Health & Social Equity, Murdoch University, 90 South Street, Murdoch, Western Australia 6150 Australia

**Keywords:** Racism, Aboriginal, Mental health, Social determinants of health, Australia

## Abstract

**Background:**

Racial discrimination is acknowledged as a central social determinant of Australian Aboriginal and Torres Strait Islander (hereafter referred to as Aboriginal) health, although quantitative empirical literature on the impacts of racism on Aboriginal children remains sparse. We use a novel, longitudinal dataset to explore the relationship between caregiver-perceived racism exposure and a range of mental health and related behavioural and physiological outcomes in childhood.

**Method:**

The study cohort comprised 1759 Aboriginal children aged 4–12 years from waves 2–8 (2009–2015) of the Footprints in Time: The Longitudinal Study of Indigenous Children (LSIC) dataset. We examined exposure to caregiver-perceived racism between 4 and 11 years as a predictor for mental health and related outcomes at ages 7–12 and substance use at 10–12 years. Unadjusted models and models adjusted for remoteness, community-level and family-level socio-economic status, child age and gender were used in analysis. Multilevel logistic regression was used in all analysis.

**Results:**

In fully adjusted models, perceived exposure to racism at ages 4–11 was associated with twice the risk of negative mental health (95% CI: 1.3–3.0), sleep difficulties (95% CI: 1.4–3.0), and behaviour issues at school (95% CI: 1.2–2.9), 1.7 times the risk of obesity (95% CI: 1.1–2.5), and nearly 7 times the risk of trying cigarettes (95% CI: 1.1–43.9). Increased risks were also found for being underweight and trying alcohol though estimates did not reach statistical significance. There was no evidence that racism was associated with poorer general health.

**Conclusion:**

Exposure to racial discrimination in Aboriginal children increased the risk for a spectrum of interrelated psychological, behavioural and physiological factors linked to negative mental health. Our results further affirm the importance of interventions aimed at reducing the prevalence of racial discrimination for the benefits of population health and health inequalities. The services and institutions which aim to support the mental health and wellbeing of Aboriginal children should also support interventions to reduce racism and implement accountable policies which prioritise this goal.

**Supplementary information:**

**Supplementary information** accompanies this paper at (10.1186/s12939-019-1045-8).

## Background

Racism is a central social determinant of health which contributes to the disparities in health observed between different racial and ethnic populations [1–7]. Racism continues to pervade the social systems and institutions which impact on the health and wellbeing of Indigenous populations worldwide, including Aboriginal and Torres Strait Islander (hereafter referred to as ‘Aboriginal’) populations in Australia [8]. More broadly, significant associations have been found between interpersonal racism and negative health and wellbeing outcomes in international studies across both child and adult populations [4, 9–15]. Yet the negative health effects of racism as a pervasive stressor have been shown to be stronger in children and young people when compared to adults [10, 16]. This is unsurprising given that the early years are understood to present a critical period of health vulnerability to adversity and stress [17, 18]. An increasing number of studies have examined the association between racial discrimination and health and wellbeing outcomes in children aged below 12 years in recent years, although relatively few have focused on children from Indigenous populations [12].

For Aboriginal populations in Australia, racism is a layered, diverse and – critically – persistent experience which encompasses interpersonal forms of racism encountered through verbal or behavioural gestures (e.g. name calling, abuse) and structural forms of racism prevalent throughout society (e.g. delayed medical attention, media misinformation) [19–22]. While contemporary forms of racism continue to evolve, racism experienced by Aboriginal Australians is also a historically informed experience tied to the colonisation of Australia by white settlers in the eighteenth Century and the subsequent social marginalisation, loss of sovereignty and land dispossession associated with this to the current day [23, 24]. Additionally, the effects of phenomena which exist at the intersection of racism and colonisation, such as intergenerational trauma, are increasingly being recognised and understood to have a profound impact on Aboriginal health [23, 25]. The common exposure to racism in the everyday life of Aboriginal children is well documented, both as direct exposure to discrimination or unfair treatment and as vicarious exposure through caregivers or family members [24]. A growing body of literature has also reported associations between racism exposure and health outcomes within this population [26–31]. Despite this nascent literature, evidence surrounding the specific impact of racism on children’s health as they transition into adolescence is currently limited.

Racism likely acts on health and wellbeing through increasing allostatic load and associated physiological dysregulation, initiating maladaptive psychological or behavioural responses or prompting changes in health behaviour [9, 12, 15]. Each of these processes contribute to the biological embodiment of racism exposure, encompassing the pathogenic pathways induced specifically by interpersonal racism as a psychosocial stressor [11, 32]. Within this context, racial discrimination exposure has been found to be more consistently and strongly associated with mental health than physical health outcomes across the life span, including during childhood and adolescence [9, 12, 15, 33, 34]. The relative strength of this association in children and adolescents may be due in part to an earlier onset of psychological over physiological effects as the psychological harm occasioned by racial discrimination exposure is more immediately apparent than any indicators of physiological harm, including metabolic or hormonal changes [35, 36]. A closer examination of mental health indicators adversely affected by experiences of racism during childhood will assist in understanding this association. This is particularly relevant for Aboriginal children as broader aspects of mental health and related risk factors such as problem behaviour and substance use have yet to be examined in this population.

This paper aims to address this knowledge gap, with a focus on Aboriginal children aged 4–12 years using data from the Longitudinal Study of Indigenous Children. We include measures of socioemotional problems, sleep difficulties, school notifications of behaviour issues and substance use within our analysis alongside weight status and general health. We expected that Aboriginal children exposed to caregiver-perceived racial discrimination would have an increased risk of negative mental health and related risk factors compared to children who have never been exposed.

## Methods

Footprints in Time: The Longitudinal Study of Indigenous Children (LSIC) is a prospective national longitudinal cohort study of Aboriginal and Torres Strait Islander children designed to capture their socioeconomic and cultural background and track their development across several domains. This study has been described more fully elsewhere [37], however in brief, LSIC used a multi-stage clustered sampling method across 11 geographic sites to recruit a non-representative national sample of Aboriginal children across two age cohorts. LSIC aimed to recruit 5–10% of the total Australian population of Aboriginal children; recruitment targeted two age-based cohorts, children aged 0.5–2 years (younger cohort) and children aged 3.5–5 years (older cohort) at baseline (2008). The survey conducts annual face-to-face interviews with the study child, their primary caregiver and their teacher. The sample totalled 1671 children at baseline and a further 88 children were recruited at wave 2, leading to a sample of up to 1759 children. To date, data for 10 waves have been collected with data from 8 waves currently available for analysis.

### Participants

Participants in the current study include children from the first 8 waves of LSIC, comprising 1759 respondents aged 6 months to 12 years. The younger cohort included 1010 children aged 6 months to 9 years and the older cohort included 749 children aged 4–12 years. By wave 8, 1255 participants provided data (75% retention). At wave 1 the mean age was 2.4 years in the full sample (SD = 1.6; age range 0–6 years), 1.1 years in the younger cohort (SD = 0.5; age range 0–2 years) and 4.1 years in the older cohort (SD = 0.5; age range 3–6 years). By wave 8 the mean age was 9.3 years in the full sample (SD = 1.5; age range 7–12 years), 8.1 years in the younger cohort (SD = 0.5; age range 7–9 years) and 11.1 years in the older cohort (SD = 0.5; age range 10–12 years). There were 887 (50.4%) males and 872 (49.6%) females in the full sample, including 510 (50.5%) males and 500 (49.5%) females in the younger cohort and 377 (50.3%) males and 372 (49.7%) females in the older cohort. Overall, 1534 (87.2%) study children were Aboriginal, 117 (6.7%) were Torres Strait Islander and 108 (6.1%) were both Aboriginal and Torres Strait Islander.

### Measures

#### Predictor variable

LSIC includes a carer-reported measure of racial discrimination asked at different waves throughout LSIC which differed by cohort. Exposure to racial discrimination was recorded from 3 to 8 years (waves 4–7) in the younger cohort and 4–11 years (waves 2–7) in the older cohort. Throughout waves 2–7 of LSIC, racism experienced by study children was reported by the primary carer in response to a question on whether the study child had been bullied or treated unfairly at preschool or school by children or adults because they were Aboriginal. Carers were able to indicate that the study child was bullied by other children, treated unfairly by adults or both. This measure was coded as a binary variable for analysis (‘Yes, bullied [kids being mean to him/her]’, ‘Yes, treated unfairly [adult being mean to him/her]’ or ‘Yes, both bullied and treated unfairly’ compared with ‘No’). In wave 8 the structure of the question was changed slightly so that the primary carer was first asked whether the study child had been bullied or treated unfairly at school and if they responded positively to this question (study child was bullied, treated unfairly or both), they were then prompted to indicate whether this was because the study child is Aboriginal or Torres Strait Islander. For the purposes of this study, carer responses of ‘always for this reason’ or ‘sometimes for this reason’ were categorised as a child that had been bullied or unfairly treated due to being Aboriginal or Torres Strait Islander.

#### Outcome variables

Health outcomes have been grouped into mental health and behaviour issues, substance use and physical health. All health outcomes were transcribed as binary variables for analysis. All outcomes were defined solely by data from wave 8, except sleep difficulties which was last asked in wave 7.

##### Mental health and behavioural issues

Measures of socioemotional problems, sleep difficulties and behavioural issues at school were the indicators of mental health and behaviour examined within this study. We chose all measures related to mental health available within the LSIC after considering completeness and timing of data collection. The Strengths and Difficulties Questionnaire (SDQ) was used to measure socioemotional problems based on primary carer reports. The SDQ is a behavioural screening questionnaire designed for use with those aged 4–17 years to identify both positive and negative emotional and behavioural attributes. This tool has been found to have adequate construct validity in separate populations of Aboriginal children aged 4–17 years across Australia [38, 39]. The questionnaire includes 20 items associated with emotional symptoms, conduct problems, hyperactivity and peer problems which are summed to create a total difficulties score ranging from 0 to 40. Based on recommended cut-off scores, the raw total difficulties score was categorised into abnormal (17–40), borderline (14–46) and normal (0–13) [40]. Scores of 14–40 (combining borderline and abnormal) were used to indicate an increased risk of negative mental health in this study [41]. Study children were determined to be experiencing sleep difficulties if their primary carer indicated that they usually had trouble getting to sleep or staying asleep over the past month. Study children were considered to have behavioural issues at school if their primary carer indicated that they had been contacted by the study child’s school due to behaving badly at school in the last twelve months.

##### Substance use

Primary carers of study children from the child cohort were asked in wave 8 to give permission for interviewers to ask study children directly about whether they had ever tried cigarettes or alcohol. Responses were categorised as having used cigarettes or alcohol if they indicated ‘yes, just a few puffs/yes, just a few sips’, ‘yes, a few times’ or ‘yes, lots of times’ in response to the questions ‘Have you ever tried cigarettes?’ or ‘Have you ever tried alcohol?’

##### Physical health

Physical health outcomes included indicators of both general health and weight status. These indicators were chosen as they are implicated with mental health. General health encompasses both physical health and socioemotional wellbeing, while childhood psychosocial factors and mental health disorders have been associated with obesity both during childhood and in later life [42–44]. Study children were determined to have high general health when the primary carer indicated that the study child’s health was generally ‘excellent’, ‘very good’ or ‘good’ and low general health when the response was ‘fair’ or ‘poor’. Children’s height and weight were translated into Body Mass Index (BMI)-for-age z-scores using the World Health Organisation (WHO) Anthro and WHO Anthro Plus programs. Children were classified as obese or underweight according to WHO and International Obesity Taskforce cut-off points for BMI-for-age z-scores [45].

#### Covariates

Factors considered to confound the association between racial discrimination and mental health were included as covariates in analytic models. These factors included geographic remoteness, community-level and family-level socioeconomic status, and the study child’s age and gender.

##### Community variables

Geographic characteristics in LSIC are reported using wave 1 data for the Level of Relative Isolation (LORI) measure, an index for remoteness levels within Australia developed based on an extension of the Accessibility/Remoteness Index of Australia. Five categories of isolation are derived; none (e.g. metropolitan areas), low, moderate, high and extreme (e.g. remote communities). Within LSIC, respondents in the ‘high’ and ‘extreme’ category have been collapsed into one item for ‘high/extreme isolation’ due to small sample size. The Index of Relative Indigenous Socioeconomic Outcomes (IRISEO) was used as a proxy measure of community-level socio-economic outcomes. IRISEO is a composite, rank order variable derived from information on the employment, education, income and housing characteristics of Aboriginal peoples from Indigenous Areas across Australia [46]. IRISEO data from wave 1 of LSIC has been categorised into quintiles for this analysis.

##### Primary carer variables

All LSIC primary carers in waves 1 and 2, alongside all new primary carers in waves 3 and 4, were asked to indicate their highest completed qualification. This measure was coded into a four-item variable: university-level education, certificate or post-school qualifications, completion of Year 11 to Year 12 or equivalent, and lower levels of completion. A binary ‘financial difficulty’ variable was defined if they answered, ‘We run out of money before payday’, ‘We are spending more money than we get’, ‘We have just enough money to get us through to the next pay day’ or ‘There’s some money left over each week but we just spend it’; a response of ‘We can save a bit every now and then’ or ‘We can save a lot’ were coded as no financial difficulty. Responses taken at wave 1 are included in this analysis. In wave 3, all primary carers were asked whether there had been times in the last five years when they did not have any place to live; ‘prior experience of homelessness’ was indicated for those that responded ‘Yes, many times’, ‘Yes, a few times’ or ‘Yes, once’.

##### Child variables

Each study child’s primary carer reported the study child’s age and gender at each wave. Child age at wave 8 was collapsed into three categories: 7–8, 9–10 and 11–12 years.

### Data analysis

Chi-squared was used to examine bivariate associations and Kendall’s Tau was used to assess potential collinearities between perceived racism and the covariates. Following this, two logistic regression models, using the generalised linear mixed modelling (GLMM) framework, were defined and fitted: a model adjusted for child age and gender (model 1) and a fully adjusted model (model 2) including child age and gender, geographic remoteness, area-level socioeconomic status and family-level socioeconomic status (primary carer reported highest education completed, financial difficulty and prior homelessness). A randomised cluster variable was included within LSIC to identify respondents living in close geographic proximity [45], this variable was developed to overcome bias introduced by the sampling method; this was the grouping variable used in the random effect modelling. Models were fitted using PROC GLIMMIX in SAS (version 9.4, SAS Institute Inc., Cary, NC, USA, 2002–12) with compound symmetry as the covariance structure. Adaptive Gaussian quadrature methods were chosen as they have been shown to perform well with excellent coverage probabilities and minimal bias when estimating the fixed effects of a GLMM for binary outcome clustered data where there are sufficient numbers of observations per random effect [47].

#### Missing data

Non-responses (missing data) were present in the predictor, outcome and covariate variables. As a sensitivity analysis, multiple imputation was used to determine the influence of missing data on parameter estimates. Two auxiliary variables (cohort and number of major events experienced in the last year) were used to assist with the prediction of missing data. Multilevel multivariate normal imputation with joint modelling was then carried out, generating 100 imputed datasets. Adaptive rounding [48] was pursued on imputed datasets as model variables were binary or categorical, however this did not return realistic data in all cases. Instead, linear mixed effects models were run on unrounded data across all imputed datasets, and combined parameter estimates were compared against the complete cases analysis (see Additional file 1). These analyses were conducted using the ‘mitml’ package [49] in R (Version 1.2.1335 –© 2009–2019).

## Results

One in five (20.4%) study children from LSIC between the ages of 4–11 years experienced at least one exposure to caregiver-perceived racial discrimination at school from either adults or peers. Close to one in six (17%) children in LSIC aged 7–12 were found to be at an increased risk of negative mental health or had behavioural issues at school reported to their primary carer, while 21% were reported to have experienced difficulty sleeping. Around one in twenty (4%) children were found to be underweight by wave 8 and 18% were obese, while 3% were identified as having fair or poor global health. Among responding children aged 10–12 years, 3% reported that they had tried smoking and 11% had tried alcohol.

After adjustment for all study variables, study children living in the most disadvantaged areas (IRISEO; quintile 1) had twice the odds of being exposed to caregiver-perceived racial discrimination at school compared with those living in the most advantaged areas (OR 2.1; 95% CI: 1.0–4.3) (Table 1). A similar effect size (OR 2.3; 95% CI: 1.4–3.7) was evident for children whose primary carer had experienced homelessness in the past five years relative to those with no experiences of homelessness. No other socio-demographic factors were associated with caregiver-perceived racial discrimination exposure.

**Table 1:** Multivariate associations between caregiver-perceived racial discrimination and selected socio-demographic characteristics in Aboriginal children

Variables		Child ever exposed to being bullied or treated unfairly because they were Indigenous		
	% (n)	Adjusted OR^a^	95% CI	*P*-value
Gender of child				
Male	50.4 (887)	Ref		
Female	49.6 (872)	1.09	0.80–1.48	0.61
Age of child (years)				
7–8	46.8 (823)	Ref		
9–10	14.2 (249)	0.91	0.56–1.47	0.73
11–12	39.1 (687)	1.13	0.82–1.56	0.32
Level of relative isolation				
None	26.0 (433)	Ref		
Low	49.4 (822)	0.75	0.50–1.14	0.20
Moderate	15.2 (253)	0.88	0.48–1.59	0.77
High	9.4 (157)	1.21	0.63–2.31	0.58
Index of relative Indigenous socioeconomic outcomes				
Quintile 5 – most advantaged	14.2 (237)	Ref		
Quintile 4	14.7 (245)	1.04	0.60–1.82	0.87
Quintile 3	41.3 (690)	1.09	0.63–1.86	0.80
Quintile 2	16.9 (282)	1.52	0.84–2.75	0.17
Quintile 1 – most disadvantaged	13.0 (217)	2.10	1.03–4.27	0.04
Primary carer highest education completed				
Never attended school to Year 10	42.6 (649)	Ref		
Year 11/12	33.5 (510)	1.26	0.88–1.80	0.18
Diploma or Certificate	18.1 (275)	1.44	0.94–2.19	0.09
University	5.8 (89)	1.26	0.63–2.55	0.45
Financial difficulty				
Save a lot/save a bit every now and then	35.5 (569)	Ref		
Some money left/just enough to get through	46.5 (745)	1.32	0.93–1.86	0.13
Spending more than we get/run out of money	18.1 (290)	1.46	0.85–2.07	0.21
Primary carer experienced homelessness				
No	91.3 (1279)	Ref		
Yes	8.7 (122)	2.30	1.42–3.71	< 0.001

^a^Odds ratios are adjusted for all variables that appear in the table

Overall, positive associations were seen between exposure to caregiver-perceived racism and mental health, behavioural issues and substance use (Table 2). In fully adjusted models, children exposed to perceived racial discrimination had around twice the risk for negative mental health, sleep difficulties, behaviour issues at school and trying alcohol (ORs ranging 1.8 to 2.0), although estimates for trying alcohol were not statistically significant (95% CI: 0.8–4.2). While estimates for children trying cigarettes were imprecise, these children were at seven times increased risk of trying cigarettes with racism exposure in the fully adjusted model (95% CI: 1.1–45.0).

**Table 2:** Models examining association between caregiver-perceived racial discrimination and mental health related outcomes in Aboriginal children

	Model 1^b^			Model 2^c^		
	Adjusted OR	95% CI	*P*-value	Adjusted OR	95% CI	*P*-value
Negative mental health						
No exposure	Ref			Ref		
Ever exposed	1.66	1.17–2.36	0.01	1.99	1.32–2.99	0.001
Sleep difficulties						
No exposure	Ref			Ref		
Ever exposed	1.85	1.31–2.63	< 0.001	2.00	1.35–2.96	< 0.001
Behaviour issues at school						
No exposure	Ref			Ref		
Ever exposed	1.68	1.15–2.44	0.01	1.88	1.21–2.91	0.01
Tried cigarettes^a^						
No exposure	Ref			Ref		
Ever exposed	4.74	1.27–17.69	0.02	7.05	1.10–45.02	0.04
Tried alcohol^a^						
No exposure	Ref			Ref		
Ever exposed	1.46	0.71–3.02	0.30	1.81	0.78–4.20	0.17
General health						
No exposure	Ref			Ref		
Ever exposed	0.71	0.26–1.94	0.50	0.70	0.23–2.12	0.53
Underweight						
No exposure	Ref			Ref		
Ever exposed	1.76	0.84–3.69	0.14	1.61	0.66–3.93	0.29
Obesity						
No exposure	Ref			Ref		
Ever exposed	1.76	1.23–2.52	0.002	1.68	1.12–2.53	0.01

^a^Child cohort only

^b^Model adjusted for child age and gender

^c^Model adjusted for child age and gender, geographic remoteness, area-level socioeconomic status and family-level socioeconomic status (primary carer reported highest education completed, financial difficulty and prior homelessness)

Positive associations were found between caregiver-perceived racial discrimination and physical health outcomes, with the exception of general health (Table 2). After adjustment for child age and gender, study children had 1.8 times the risk of experiencing obesity (95% CI: 1.2–2.5)—with a similar odds ratio in the fully adjusted model (OR: 1.7; 95% CI: 1.1–2.5)—compared with children with no exposure. A moderate effect for being underweight was also observed in the fully adjusted model (OR: 1.6; 95% CI: 0.7–3.9), although the effect estimate was not statistically significant. A negative association was found for general health though this did not reach significance.

Socio-demographic factors were also included in logistic regression models with each health outcome. After adjustment for all covariates, the following covariates and health outcomes were found to be significantly associated. Compared to males, females had lower odds for negative mental health (OR: 0.6; 95% CI: 0.4–0.9), behaviour issues at school (OR: 0.2; 95% CI: 0.1–0.3) and having tried alcohol (OR: 0.2; 95% CI: 0.1–0.4). Children living in the most disadvantaged areas had lower odds of obesity compared to those living in the most advantaged areas (OR: 0.2; 95% CI: 0.1–0.9). Living in areas of moderate remoteness (i.e. regional towns) was associated with lower odds of sleeping difficulties (OR: 0.3; 95% CI: 0.1–0.7) compared to living in the least remote areas (i.e. cities). Children at an older age (11–12 years) were significantly more likely to have negative general health than those aged 7–8 years (OR: 2.4; 95% CI: 1.0–6.0). Children had decreased odds of socioemotional difficulties where primary carers had completed Year 11 or 12 (OR: 0.6; 95% CI: 0.4–0.9) or university (OR: 0.3; 95% CI: 0.1–0.9) compared to primary carers who had completed Year 10 education or less.

Analysis using imputed datasets resulted in pooled estimates for each outcome that were similar to estimates from complete case analysis. Pooled estimates fell within confidence intervals from complete case analysis and confidence intervals from both analyses overlapped considerably. All significant associations between racial discrimination and health outcomes found in complete case analysis were also found in analysis of imputed datasets.

## Discussion

This study sought to broadly assess the effects of racial discrimination exposure on mental health in Aboriginal children. We found that Aboriginal children exposed to racial discrimination between ages 4–11 were at higher risk of negative mental health and related behavioural and physical outcomes compared to children without racism exposure. Children with caregiver-perceived racism exposure had around twice the risk of negative mental health, sleep difficulties, behavioural issues at school and obesity.

In a novel finding, we saw that Aboriginal children exposed to racism had seven times the risk of trying cigarettes at ages 10–12 compared to unexposed children. The strong, albeit imprecise, effect of racial discrimination on trying cigarettes found in our study is reflected in longitudinal studies conducted in both the United States and United Kingdom examining childhood exposure to racism as a predictor of later smoking behaviour, though both studies measured smoking behaviour in adulthood (21–25 years) [50, 51]. Studies in child (6–11 years) and adolescent (12–18 years) populations are broadly mixed, with around half of studies finding a significant positive association between racism and smoking [12]. While our study focused on racism exposure during childhood, the positive associations we found between racism and both trying cigarettes and alcohol reflect those seen between trajectories of racial discrimination throughout adolescence and later risk of substance use (encompassing alcohol, tobacco and marijuana use) [52–55]. It is likely that racism exposure, substance use and mental health disorders are interrelated as the onset of mental health disorders in childhood known to be strongly associated with racial discrimination, such as anxiety and conduct disorder, is predictive of later substance use [56] and substance use disorder [57]. The association between racial discrimination and cigarette smoking in Aboriginal children warrants further examination, particularly in older cohorts and longitudinally, to fully assess the nature and size of this association by age and over time.

We further confirmed the broad reaching effects of racial discrimination on mental health through the strong and consistent associations found between racism and socioemotional, sleep and behavioural difficulties in this cohort of Aboriginal children. Strong associations between racism and negative mental health and behavioural issues during this developmental period are supported in the literature, with positive associations found in children aged 6–11 years [12] and under 13 years [10]. Significant associations have also been found between racial discrimination and sleep difficulties both in cross-sectional [58, 59] and longitudinal studies [60, 61], although these are predominantly based on adolescent and adult populations [62]. Behavioural problems, negative mental health and sleep difficulties are known to be associated both concurrently and longitudinally [63–65]. Racial discrimination exposure may contribute to the development of these related factors as a psychosocial stressor by initiating the emotional and psychological responses which characterise negative mental health symptomology [66]. Findings from this study underscore the reality that racial discrimination is a central social determinant of the mental health of Aboriginal children. Interventions, policies and practices designed to recognise and address racial discrimination in this population must be considered by the programs, services and institutions which aim to support the mental health and wellbeing of Aboriginal children, particularly within the healthcare and education sectors.

Changes in weight status found in Aboriginal children exposed to racism reflect those seen for socioemotional, behavioural and substance use outcomes within our analysis. We found that children exposed to caregiver-perceived racial discrimination had an increased risk of obesity and being underweight, though estimates for being underweight did not reach significance. The positive association found between perceived racial discrimination and obesity in our study is supported by findings from both child [27] and adolescent [67] populations, though the majority of studies in child populations have found no association [12]. This may be due in part to the complexity of processes underlying the association between physiological responses to stress and obesity or metabolic syndrome [68–70]. Yet there is evidence that mental health plays an important role in mediating and modifying the effect of racial discrimination on BMI during adolescence [67, 71]. One longitudinal study reported an indirect association between racial bullying and increased BMI through greater negative emotional symptoms [67] while another found that high protective emotional support from parents attenuated BMI in adolescents who perceived racism [71]. While our findings confirm a direct association between racism and weight status, this may be complicated by unexamined mediating or moderating pathways. Future studies should consider potential mediating and moderating factors in the association between racial discrimination and weight status in Aboriginal children to better clarify the function of mental health within this relationship.

Although this study extended previous research on the association between racism and the mental health of Aboriginal children, our findings should be interpreted in light of several limitations. The instrument used to measure racism exposure in this study was based on the perceptions of primary carers rather than self-reported by study children. Although primary carers reported on the experiences of study children to the best of their knowledge, these reports may not necessarily reflect the perceptions of racism in these children. Additionally, the racism exposure instrument did not specify a timeframe for racism exposure or quantify the frequency or severity of racism exposure. These factors reduce the sensitivity of this instrument as children with many different experiences with racism were unable to be distinguished from one another. Finally, despite the strength of the LSIC as a prospective cohort study with strong community involvement, the sampling design was non-random due to practical and logistic considerations in site selection [72]. As a result, the sample of study children in LSIC is not representative of the population of Aboriginal children across Australia. However, the sites selected for the study were intended to reflect the geographic distribution of Aboriginal children within Australia and the varied environments in which these children live [45].

## Conclusions

Exposure to racial discrimination in Aboriginal children increases the risk for a spectrum of interrelated psychological, behavioural and physiological factors encompassed by mental health. This includes an increased risk of socioemotional and behavioural difficulties, changes in weight status and, in a novel result, substance use. Findings from this study contribute to the limited body of evidence surrounding the contribution of racial discrimination towards mental health and wellbeing outcomes in Aboriginal children. Initiatives to reduce the prevalence of racism may act to simultaneously attenuate the risk for a range of factors related to mental health. These initiatives must be driven by the Aboriginal populations involved (from inception to delivery) to ensure they are culturally relevant and draw from Aboriginal voices and paradigms [21]. Aboriginal adults have identified that addressing racism involves both internal and external processes, including distancing oneself from racism and strengthening a sense of identity and pride in being Aboriginal while emphasising the need for society to acknowledge racism and challenge racist attitudes and behaviours [20]. Given that racial discrimination is an ongoing public health concern for Australian Aboriginal children, school-based interventions informed by the above parameters would be well placed to address the effects of this critical social determinant and target a reduction in its prevalence.

## Supplementary information

**Additional file 1:** Comparison of estimates from imputation and complete case analysis. (DOCX 16 kb)

## Data Availability

The dataset analysed in this study is available, on request, from the Australian Government Department of Social Services via the Australian Data Archive Dataverse platform: https://dataverse.ada.edu.au/dataverse/ncld**.**

## Abbreviations

BMI: Body Mass Index
CI: Confidence Interval
GLMM: Generalised Linear Mixed Model
IRISEO: Index of Relative Indigenous Socioeconomic Outcomes
LORI: Level of Relative Isolation
LSIC: Longitudinal Study of Indigenous Children
OR: Odds Ratio
SD: Standard Deviation
SDQ: Strengths and Difficulties Questionnaire
SEIFA: Socioeconomic Index for Areas
SES: Socioeconomic Status
WHO: World Health Organisation
