# Supplementary material for: Caregiver-perceived racial discrimination is associated with diverse mental health outcomes in Aboriginal and Torres Strait Islander children aged 7–12 years
Source: Int J Equity Health. 2019 Sep 6;18:142. doi: 10.1186/s12939-019-1045-8 (PMC6729036; doi:10.1186/s12939-019-1045-8)
Supplement: Supplementary file 1 — Additional file 1: Comparison of estimates from imputation and complete case analysis. (DOCX 16 kb) [file 12939_2019_1045_MOESM1_ESM.docx]

Multiple imputation and adapting rounding

Multiple imputation using mixed effects models is a relatively novel procedure and best practice recommendations are currently limited. Based on this limited literature, adaptive rounding of ordinal and binary variables returned from linear models using the ‘mitml’ package in R Studio was originally pursued in this analysis. First, ordinal variables were recoded into a series of dummy variables, these were then entered into linear mixed effects models for each health outcome. Adaptive rounding based on Bernaards et al. calculation for a cut-off score based on normal approximation to the binomial distribution was then applied to all variables. Ordinal variables were then recreated based on the dummy variables originally derived from each variable. However, due to the rounding procedure, cases would occasionally be returned where all levels of the ordinal variable were rounded to zero or more than one level of the variable was rounded to 1. This led to unrealistic cases where, for example, individuals were not allocated to any remoteness level or were allocated into multiple remoteness levels at once. Resolving this required implementing alternate or multiple rounding procedures, such as naïve rounding, to ensure that each case was allocated into a single category within ordinal variables. This process would likely lead to biased estimates and so we chose not to pursue any rounding procedure. Although the strategy of no rounding meant that continuous data was used in analysis, not the binomial data originally used with complete-case analysis, we preferred to treat both sets of data as continuous rather than introduce bias through rounding. To ensure estimates were comparable we contrasted the pooled estimates from linear models run on the imputed continuous data with linear models run on the complete-cases data.

Comparison of estimates from imputation and complete case analysis

|  | Imputation^[[1]](#footnote-1)^ | Complete cases |
| --- | --- | --- |
|  | β (SE) | β (SE) |
| **Negative mental health** |  |  |
| No exposure | Ref | Ref |
| Ever exposed | **0.08 (0.03)** | **0.10 (0.03)** |
| **Sleep difficulties** |  |  |
| No exposure | Ref | Ref |
| Ever exposed | **0.10 (0.03)** | **0.10 (0.03)** |
| **Behaviour issues at school** |  |  |
| No exposure | Ref | Ref |
| Ever exposed | **0.07 (0.03)** | **0.08 (0.03)** |
| **Tried cigarettes^** |  |  |
| No exposure | Ref | Ref |
| Ever exposed | **0.04 (0.02)** | **0.05 (0.02)** |
| **Tried alcohol^** |  |  |
| No exposure | Ref | Ref |
| Ever exposed | 0.03 (0.04) | 0.06 (0.04) |
| **General health** |  |  |
| No exposure | Ref | Ref |
| Ever exposed | -0.01 (0.01) | -0.01 (0.01) |
| **Underweight** |  |  |
| No exposure | Ref | Ref |
| Ever exposed | 0.03 (0.01) | -0.02 (0.01) |
| **Obesity** |  |  |
| No exposure | Ref | Ref |
| Ever exposed | **0.09 (0.03)** | **0.07 (0.03)** |

^Child cohort only

1. Models adjusted for child age and gender, geographic remoteness, area-level socioeconomic status and family-level socioeconomic status (primary carer reported highest education completed, financial difficulty and prior homelessness). [↑](#footnote-ref-1)
